# Modeling the greenhouse gas emissions of an immunization program against respiratory syncytial virus in infants in the United Kingdom

**DOI:** 10.1017/S0266462325100524

**Published:** 2025-10-08

**Authors:** Richard D.A. Hudson, Thierry Rigoine de Fougerolles, Flora Leadley, Mersha Chetty, Priscille De La Tour

**Affiliations:** 1 Health Outcomes, Sanofi, Reading, UK; 2 CVA, Paris, France; 3 CVA, London, UK; 4 Environment & Health, Sanofi, Paris, France

**Keywords:** carbon dioxide emissions, sustainability, care pathway, immunization, prevention

## Abstract

**Objectives:**

The healthcare system accounts for 4 percent of United Kingdom (UK) greenhouse gas (GHG) emissions annually. In response to climate change, the National Health Service (NHS) is calling for less carbon-intensive care practices through prevention. Respiratory Syncytial Virus (RSV), a leading cause of infant hospitalization, currently has no widespread immunization program in the UK. This study estimates the impact on GHG emissions generated within the care pathway from an immunization against RSV in all infants in the UK with nirsevimab, a new monoclonal antibody used in prophylaxis.

**Methods:**

A novel approach was applied, mapping care pathway emissions from immunization and avoiding RSV-related primary and secondary care burden. Avoided healthcare resources were estimated using a published health economic model for nirsevimab versus standard of care (SoC), which is characterized as receiving palivizumab or having no immunization intervention, assuming different universal immunization scenarios. NHS England GHG emission factors were applied to each health outcome to measure the GHG emissions associated with a nirsevimab versus SoC strategy.

**Results:**

Compared with SoC, a universal immunization program using nirsevimab leads to avoided GHG emissions, amounting to ~22 kilotons of CO_2_ equivalents per year, with immunizing all UK infants at birth leading to the greatest reduction. About 40 percent of avoided emissions were from reductions in inpatient hospitalizations.

**Conclusions:**

This study shows how prevention can deliver benefits to people, NHS system capacity, and the environment. However, avoided patient care pathway emissions must be considered alongside drug lifecycle emissions, which are not included here.

## Background

The healthcare system accounts for around 4 percent of total United Kingdom (UK) greenhouse gas (GHG) emissions every year (1). The majority of emissions, around 62 percent, stem from the supply chain for medicines, medical devices, and consumables, while patient care, as well as patient and visitor travel to and from National Health Service (NHS) sites, account for 26 and 6 percent, respectively (1). As part of the ambition to mitigate the effects of climate change and reduce its carbon footprint (CF), the NHS has committed to achieving net zero by 2040 for its direct emissions and by 2045 for its CF “plus,” which includes scope 3, patient and staff travel emissions. Scope 3 emissions are those for which the NHS is indirectly responsible, through the impact of its value chain activities. Patient and visitor travel are not within scope 3, but were considered in the evaluation of the NHS England by Tennison *et al*. This calls for less carbon-intensive care practices and more sustainable models of care, including prevention strategies (2). Disease prevention is a key pillar among the seven levers to reduce emissions in care pathways identified by the Sustainable Markets Initiative (SMI) Health Systems Task Force and immunization programs are highlighted as having the potential to reduce GHG emissions along the patient care pathway. The task force calls for further studies to measure their impact (3).

The public health benefits of prevention are well documented, but there is limited scientific literature specifically showing how disease prevention or immunization programs might avoid GHG emissions, and few studies provide estimates of associated GHG reduction due to the programs themselves. Current approaches to assessing environmental impact often focus on measuring the GHG emissions of a system or organization, like the NHS, in the context of their GHG reduction goals (2) or on a specific product from the CF perspective covering raw material extraction, manufacturing, distribution, usage, and end of life. Recent studies, for example, on COVID-19 vaccines, have used a CF approach to estimate the GHG emissions from storage, distribution, and disposal, but have not provided estimates of GHG emissions at the drug use phase (4;5). Moreover, in the scope 3 calculation methodology, GHG emissions from the use phase are limited to those attributed to the direct use of the drug or the indirect use-phase from associated energy consumption (fuels or electricity) (6). This scope does not capture the broader effect of medicine on human health, which leads to reduced healthcare utilization, such as fewer hospital admissions. Analysis along the patient care pathway provides the scope to determine broader environmental effects of an immunization intervention without restricting the CF analysis to the product or specific stages of the scope 3 protocol. This includes the impact of positive health outcomes for the health system, namely those associated with the administration of an immunization solution, which potentially contributes to lower health system resource use. In the field of prevention, GHG emissions along the patient care pathway from the point of immunization and associated clinical outcomes remain largely unexplored.

Immunization solutions can simplify the patient journey due to fewer doses required and provide protection in populations for which no solution currently exists. This potentially leads to better outcomes, reduced healthcare utilization, and lower associated GHG emissions, contributing to the sustainability and resilience of healthcare systems (7). However, to date, there is limited progress in including environmental sustainability within health technological assessments (HTAs), although this is increasingly considered in government tenders (8). To promote more sustainable healthcare delivery, decision makers and clinicians could consider the GHG emissions of different medical treatments, and their broader environmental impacts, like water footprint, from the patient care pathway perspective, and incorporate this into medicine assessment frameworks (9). This would provide a health system-focused approach to their assessment, while life cycle assessment (LCA) and CF bring a product perspective. Among other environmental impacts like waste or particulate matter, the GHG cost of healthcare can be assessed not only with regard to the environmental impact of the product lifecycle but also to potential environmental impact along the patient care pathway from addressing health outcomes.

Respiratory syncytial virus (RSV) is a highly contagious infectious disease with no widespread immunization program in infants in the UK (10;11). RSV seasonal epidemics pose a significant public health burden and lead to substantial healthcare utilization in the UK, particularly in the winter months (12;13). Each year, RSV infections cause mild to severe health outcomes, such as lower respiratory tract diseases, bronchiolitis, or pneumonia, leading to around 467,000 visits to general practitioners (GPs) and up to 34,000 hospitalizations in the UK in children under 5 years of age (13–15). RSV is a leading cause of infant hospitalization. Hospitals are one of the highest producers of GHG emissions amongst public health settings; reducing acute care utilization has clear environmental benefits in addition to freeing up capacity (16). Similar to other respiratory viruses, RSV transmission is also influenced by climate conditions, among other factors, resulting in a possible shift in geographic distribution and variability of seasonal cycles due to varying weather conditions (17). The World Health Organization (WHO) recognizes the need for a broader RSV prevention strategy that extends protection to all infants (18) and the Joint Committee on Vaccination and Immunization (JCVI) issued an opinion supporting a universal immunization program against RSV in all infants (19). AstraZeneca has developed a single-dose, long-acting monoclonal antibody (mAb), nirsevimab, for passive immunization against RSV-related lower respiratory tract infection (RSV LRTI) for infants facing their first RSV season. Commercialized by Sanofi, nirsevimab was licensed by the Medicines and Healthcare products Regulatory Agency on 9^th^ November 2022 (20) and in July 2023, the JCVI issued an opinion that nirsevimab could be considered to prevent RSV LRTI in all infants under the age of 1 year (19).

This exploratory study aims to estimate patient care pathway GHG emissions associated with the introduction of a universal immunization program to administer nirsevimab to all infants under 1 year of age to prevent RSV LRTI.

## Methods

### Scope

This study focuses on immunization against RSV-related LRTI in infants under 1 year of age in the UK, aligned to the recent JCVI opinion. The analysis compares the incremental environmental benefits of an intervention with nirsevimab to standard of care (SoC), which is characterized as an intervention with the administration of palivizumab, currently used in approximately 4,000 high-risk infants, and no immunization intervention in infants not eligible for palivizumab, as per recommendations (11). A universal immunization program population corresponds to around 712,000 eligible individuals, utilizing the UK 2019 pre-pandemic birth cohort size (21–23). The functional unit is 1 dose of nirsevimab, administered using a single-use injection device for 1 eligible UK patient (24–26). The study evaluates the impact on GHG emissions of the patient care pathway, which includes administration and the drug’s therapeutic impact on GHG emissions. GHG emissions associated with material acquisition and pre-processing, production, distribution, and storage, and end of life of nirsevimab were excluded as CF data were not yet available at the time of the study.

### Patient care pathway approach

**Figure 1.**
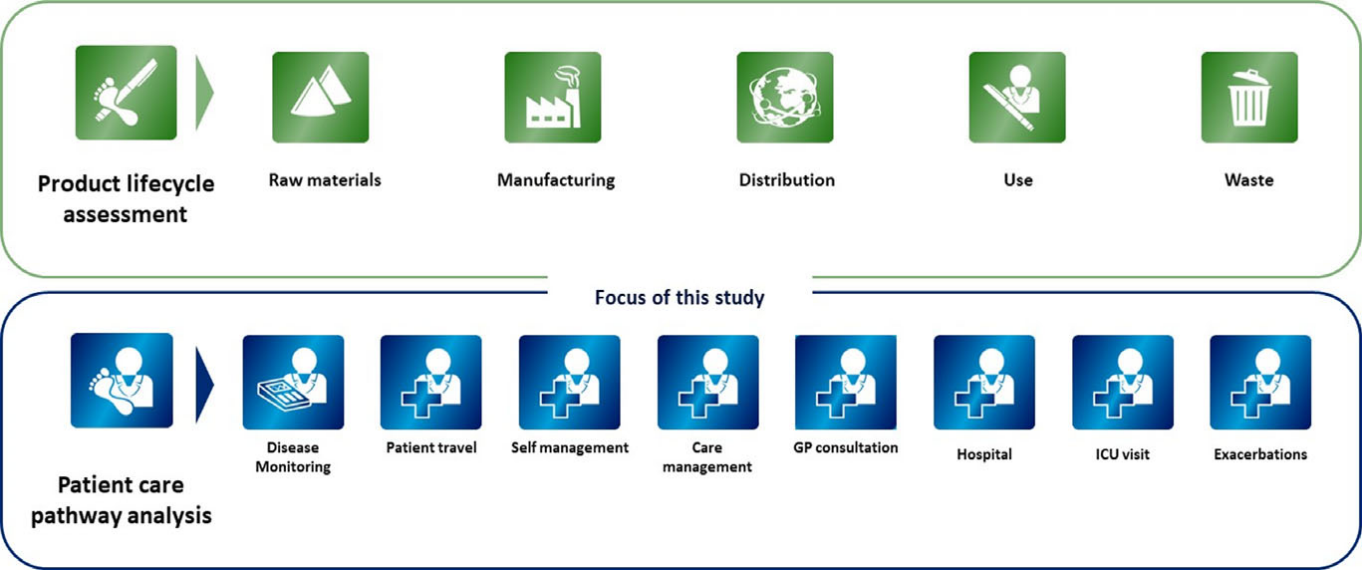
Schematic comparing product life cycle assessment and patient care pathway analysis. Source: Sustainable Healthcare Coalition. Available from https://shcoalition.org/sustainable-care-pathways-guidance/. Accessed 29 July 2025.

**Figure 2.**
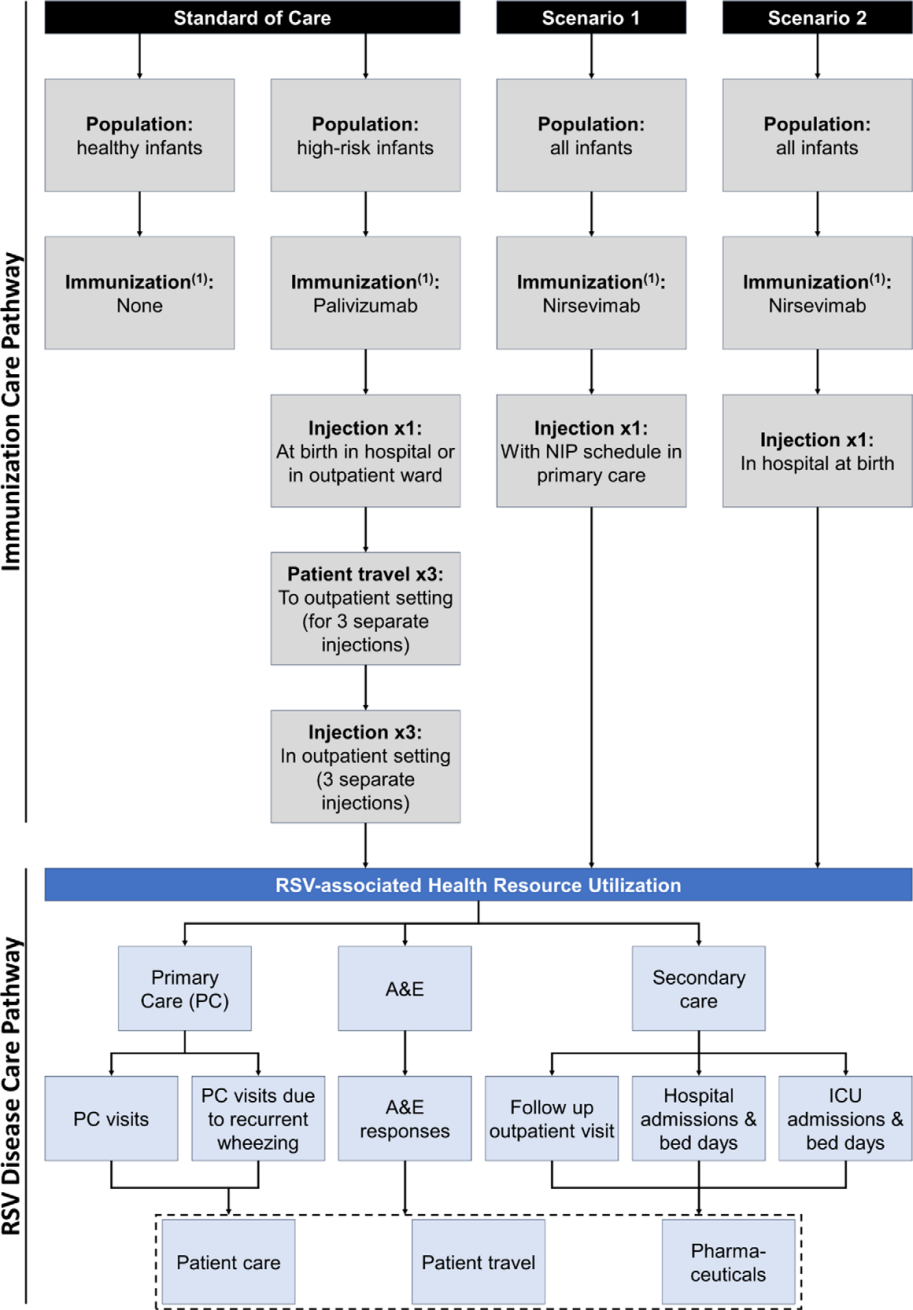
Patient care pathways for palivizumab and nirsevimab immunization. *Note*: (i) immunization includes the materials used during the immunization process (use of ethanol, cotton and bandages for each injection) and excludes the GHG footprint of the product accident and emergency (A&E), average (avg.), general practitioner (GP), intensive care unit (ICU), National Immunisation Programme (NIP), primary care (PC). (ii) product manufacturing, distribution, and disposal emissions are excluded from this analysis.

The patient care pathway mapping was conducted in line with technical guidance on sustainable care pathways issued by the Sustainable Healthcare Coalition (27) (see Figure 1 for a comparison of LCA and patient pathway analysis). A theoretical care pathway was formalized, aligned with the published health economic model that provides the input for this study (28). The selected care pathway modules for RSV disease are: GP consultation (primary care visits), emergency department visit (accident & emergency visit, outpatient visit), inpatient/bed day (inpatient hospitalization, intensive care unit), and patient travel, while surgical procedure and condition self-management were excluded (Figure 2). A consultation with clinical experts in an advisory board confirmed the selection of care pathway modules. No audit of clinical pathways was performed. The mapping covers emissions from both the patient journey to get immunized (Table 1), including patient travel to the immunization setting where relevant, and the avoided RSV health outcomes within primary and secondary care as a result of immunization. The approach excludes other activities at the immunization setting that are not related to the administration of the immunization, which may also incur emissions.

**Table 1.** RSV disease burden and healthcare utilization in infants in the UK, as modeled for standard of care, Scenario 1 and Scenario 2

*Figures in absolute numbers*	Standard of care			Scenario 1						Scenario 2					
				Base	Lower	Upper	Base S1 -	Lower S1 -	Upper S1 -	Base	Lower	Upper	Base S2 -	Lower S2 -	Upper S2 -
	Base	Lower	Upper				Base SoC *(that is avoided events)*	Lower SoC *(that is avoided events)*	Upper SoC *(that is avoided events)*				Base SoC *(that is avoided events)*	Lower SoC *(that is avoided events)*	Upper SoC *(that is avoided events)*
RSV disease burden – Events															
Primary care visits	159,371	108,715	178,500	58,224	39,710	65,228	(101,147)	(69,004)	(113,272)	56,510	38,772	63,211	(102,861)	(69,943)	(115,288)
Primary care visits related to recurrent wheezing	18,286	12,474	20,476	6,301	4,307	7,054	(11,984)	(8,167)	(13,422)	5,917	4,057	6,618	(12,369)	(8,417)	(13,858)
Accident and emergency visits	64,575	64,575	64,575	24,730	24,730	24,730	(39,845)	(39,845)	(39,845)	23,044	23,044	23,044	(41,531)	(41,531)	(41,531)
Inpatient hospitalization (incl. ICU)	24,381	16,632	27,301	8,402	5,742	9,405	(15,979)	(10,890)	(17,896)	7,889	5,409	8,823	(16,492)	(11,223)	(18,478)
Intensive care unit cases	1,147	814	1,272	396	282	440	(750)	(532)	(833)	375	268	415	(772)	(546)	(857)
Follow up consultant outpatient visit	1,147	814	1,272	396	282	440	(750)	(532)	(833)	375	268	415	(772)	(546)	(857)
Total	268,906	204,025	293,396	98,450	75,055	107,296	(170,456)	(128,970)	(186,100)	94,109	71,820	102,527	(174,797)	(132,205)	(190,869)

*Note*: Product manufacturing, distribution, and disposal emissions are excluded from this analysis.

Abbreviations: SoC, standard of care; S1, scenario 1; S2, scenario 2; ICU, intensive care unit.

### Immunization pathway

One injection is indicated for nirsevimab to offer protection against RSV for at least 5 months, or the length of a typical RSV season in temperate climates like the UK, typically between October and March. Two ways to deliver nirsevimab to all infants were considered: either at birth in the hospital or concomitantly with other childhood vaccinations. For infants born during the RSV season, this immunization is modeled to take place at birth in hospital and therefore does not generate incremental patient travel (Figure 2). If the infant is born outside of the RSV season, immunization is delivered concomitantly to other childhood vaccinations as part of the UK’s National Immunization Programme (NIP) and therefore is included in an existing primary care visit (11). This differs from the administration of palivizumab in the small high-risk population of eligible infants, where monthly injections are required with an average of four injections modeled. The number of injections given in reality depends on the birth date relative to the season. The first injection is either at birth in the hospital or in an outpatient setting; for the remaining follow-on injections, the infant is required to attend an outpatient setting, typically within a hospital, generating emissions from patient travel and healthcare utilization (11).

### Disease burden care pathway

To estimate GHG emissions from avoided disease burden as a result of the protection offered by nirsevimab, emission factors were applied to specific incremental health outcomes from a published United States (US) health economic model adapted for the UK (28). An all-infant immunization program was assumed with a 91 percent uptake, based on UK COVER data, considering the expected NIP implementation route (29). This equates to approximately 650,000 immunized infants (30). Avoided RSV health outcomes considered were primary care visits, primary care visits related to recurrent wheezing, accident and emergency (A&E) visits, inpatient hospitalizations, intensive care unit (ICU) cases, and follow-up consultation outpatient visits. The incremental avoided healthcare events were multiplied by the estimated unit of activity-based GHG emission factors from Tennison *et al.* using a simple static model (Table 1).

### Emissions factors

Two sources, Tennison *et al.* (1) and Penny *et al.* (27), were identified from the literature as a source for NHS England emission factors. The emissions in Tennison *et al.* were calculated using a hybrid accounting method; top-down for emissions where there is currently a lack of available bottom-up data or measured by using the monetary cost of a unit of interest to estimate environmental impact, and bottom-up for those where the quantitative measure of a level of activity that results in GHG emissions, activity data, can be measured, modeled, or calculated. The emissions factors in Penny *et al.* were calculated using an activity-based life cycle approach which includes patient travel as a separate environmental module. Of these, the emissions factors from Tennison *et al.* represent the latest NHS England unitary emission factors available from the 2019 NHS CF covering scope 1 (direct emissions occurring from sources controlled or owned by an organization), scope 2 (emissions caused indirectly by a company from the energy it purchases and uses) and scope 3 (indirect emissions not produced by a company itself or as a result of its activities or assets but those it is indirectly responsible for along its value chain), as well as an extended scope 3 including patient and visitor travel; so, this source was used for the analysis (1). The following estimates per healthcare event from Tennison *et al.* were used: 66 kg CO_2_eq per primary care visit, 75 kg CO_2_eq per A&E visit, 76 kg CO_2_eq per outpatient visit, and 125 kg CO_2_eq per bed day of hospitalization (1). No data were available per bed day of ICU, and it was decided to take the conservative approach of applying the same emission factor as for hospitalization, recognizing that the actual emissions volume may be higher due to the intensity of treatment. In the absence of equivalent data from the other UK nations, these figures were applied universally (21–23). The emissions data are not associated with a specific age range or disease.

Beyond healthcare utilization emission factors, certain factors specific to the immunization procedure itself were included, like the use of ethanol, cotton, and bandages for each injection. Given the similarities with influenza immunization, these data were extracted from an LCA for a high-dose influenza vaccine in the US, performed in line with the Product Environmental Footprint (PEF) method (31). GHG emissions associated with the use and end of life of ethanol, cotton, and bandages used for immunization were estimated as 0.02 kg CO_2_eq per injection. The emissions associated with the production, distribution, storage, and disposal of nirsevimab and palivizumab have been excluded from the assessment as they were not available at the time of the study.

### Scenarios

Two scenarios were considered, assuming different patient care pathways for immunization and duration of protection for nirsevimab. The first scenario (scenario 1 or S1) assumed infants to be immunized at birth in the hospital if born in-season or at the closest appointment to the beginning of the season, within existing NIP appointments if born out of season, with protection up to 150 days according to the licensed duration of protection for nirsevimab. The JCVI has recognized that the complete loss of efficacy after the license period of protection of 150 days is unlikely, and so, in a second exploratory scenario (scenario 2 or S2), all infants are immunized at birth in hospital with nirsevimab, regardless of birth month, with immunity waning after 150 days to 50 percent at 1 year (32). This exploratory analysis is on the basis of the South African data published as part of the evidence from the MELODY clinical trial (33) and can be supported by serum concentrations of neutralizing antibody at 1 year (34).

The inputs for this analysis, taken from the health economic model, are outlined in Table 1 and illustrate the estimated number of RSV disease-related health events in the UK every year for SoC and the two scenarios (29). In contrast to the current SoC, where no intervention is available for a majority of infants, the health economic model predicts a substantial reduction in the burden of RSV-related LRTI in infants after nirsevimab immunization, with scenario 2 (immunization at birth for all infants with waning of efficacy after 150 days) leading to the largest reduction in HCRU (56,510 RSV-related primary care visits, 23,044 A&E visits and 7,889 inpatient hospitalizations avoided). 4.5 and 6.1 bed days were used as the mean length of stay for an infant with RSV-related LRTI, in the hospital and the ICU, respectively (35–37). The health outcomes used as input in this study are generally consistent with the outcomes from the independent model used by the JCVI (38).

### Sensitivity analysis

Given the variability in the impact of RSV epidemics, a sensitivity analysis was performed on the RSV-related disease burden input. Upper and lower boundaries were based on different hospitalization rates from two published studies documenting RSV-related burden in the UK (15;36). RSV hospitalization rates in infants used were 22.4/1,000 for the lower bound and 35.1/1,000 for the upper bound, resulting in the inputs described in Table 1.

### Expert review

Given the novelty of the study, a selection of four experts was consulted to review the methodology, audit the GHG reduction model, and assess the data sources used. The expert profiles included health economic modelers, health and environment specialists, and carbon footprinting experts. During online consultations that took place from July to September 2023, the experts acknowledged the novelty of the study, confirmed its robustness and timeliness, and agreed with the limitations identified.

## Results

Compared with SoC, a universal infant RSV immunization program with nirsevimab has the potential to substantially reduce GHG emissions throughout the patient care pathway (Table 2). Current UK SoC GHG emissions were estimated at 32.9 kilotons (kt) CO_2_eq annually, of which 1.6 kt CO_2_eq was attributable to immunization emissions incurred in the palivizumab-eligible cohort and 31.3 kt CO_2_eq due to the health outcomes from RSV epidemics in the much larger cohort of healthy term and preterm infants, which remain. Assuming that protection would be limited to 150 days and the implementation of an at birth and catch-up program (scenario 1), universal RSV immunization for infants entering their first RSV season could avoid emissions of up to 21.7 kt CO_2_eq annually in the UK. Considering the limited scope of the product environmental assessment, if the protection is assumed to last longer than 150 days (scenario 2), then immunizing all infants at birth against RSV could save up to 22.3 kt CO_2_eq annually.

**Table 2.** Patient care pathway CO_2_eq for a universal RSV immunization program using nirsevimab in the UK, as modeled for Standard of Care, Scenario 1 and Scenario 2

*Figures in kt CO_2_eq*	Standard of care			Scenario 1						Scenario 2					
				Base	Lower	Upper	Base S1 -	Lower S1 -	Upper S1 -	Base	Lower	Upper	Base S2 -	Lower S2 -	Upper S2 -
	Base	Lower	Upper				Base SoC *(that is avoided emissions)*	Lower SoC *(that is avoided emissions)*	Upper SoC *(that is avoided emissions)*				Base SoC *(that is avoided emissions)*	Lower SoC *(that is avoided emissions)*	Upper SoC *(that is avoided emissions)*
**Immunization patient journey**															
Immunization GHG emissions^a^	1.61	1.61	1.61	0.02	0.02	0.02	(1.60)	(1.60)	(1.60)	0.02	0.02	0.02	(1.60)	(1.60)	(1.60)
**RSV disease burden - emissions**															
Primary care visits	10.5	7.2	11.8	3.8	2.6	4.3	(6.7)	(4.6)	(7.5)	3.7	2.6	4.2	(6.8)	(4.6)	(7.6)
Primary care visits related to recurrent wheezing	1.2	0.8	1.4	0.4	0.3	0.5	(0.8)	(0.5)	(0.9)	0.4	0.3	0.4	(0.8)	(0.6)	(0.9)
Accident and emergency visits	4.9	4.9	4.9	1.9	1.9	1.9	(3.0)	(3.0)	(3.0)	1.8	1.8	1.8	(3.2)	(3.2)	(3.2)
Inpatient hospitalization (incl. ICU)	13.7	9.4	15.4	4.7	3.2	5.3	(9.0)	(6.1)	(10.1)	4.4	3.0	5.0	(9.3)	(6.3)	(10.4)
Intensive care unit cases	0.9	0.6	1.0	0.3	0.2	0.3	(0.6)	(0.4)	(0.6)	0.3	0.2	0.3	(0.6)	(0.4)	(0.7)
Follow up consultant outpatient visit	0.1	0.1	0.1	0.0	0.0	0.0	(0.1)	(0.0)	(0.1)	0.0	0.0	0.0	(0.1)	(0.0)	(0.1)
**Subtotal**	**31.3**	**22.9**	**34.5**	**11.2**	**8.3**	**12.3**	**(20.1)**	**(14.7)**	**(22.2)**	**10.6**	**7.8**	**11.7**	**(20.7)**	**(15.1)**	**(22.8)**
***Emissions avoided per infant (kg CO***_***2***_ ***eq)***							***(31.0)***	***(22.7)***	***(34.2)***				***(31.9)***	***(23.3)***	***(35.2)***
**Net impact**	**32.9**	**24.6**	**36.1**	**11.2**	**8.3**	**12.3**	**(21.7)**	**(16.3)**	**(23.8)**	**10.6**	**7.9**	**11.7**	**(22.3)**	**(16.7)**	**(24.4)**

Abbreviations: SOC, standard of care; S1, scenario 1; S2, scenario 2; ICU, intensive care unit; CO_2_eq, carbon dioxide equivalent; kt, kiloton; kg, kilogram.

a Immunization GHG emissions refer to the materials used during the immunization process (use of ethanol, cotton and bandages for each injection) and exclude the GHG footprint of the product.

In both scenarios, no incremental patient travel to receive an immunization was assumed, given each scenario includes immunization at an existing healthcare interaction. Emissions as a result of administering the immunization were limited to the use of cotton, ethanol, and a bandage during the immunization process, amounting to 0.02 kt CO_2_eq overall. In both scenarios, administering palivizumab in high-risk infants would no longer be needed. Since palivizumab requires, on average, one injection at an existing healthcare interaction at birth and three dedicated outpatient visits, an estimated 1.6 kt CO_2_eq would be avoided at the immunization stage, representing ~8 percent of total avoided emissions.

The direct RSV-related LRTI disease burden avoided was 20.1 kt CO_2_eq in scenario 1 and 20.7 kt CO_2_eq in scenario 2, which accounted for the vast majority (~92 percent out of total emissions) of GHG emissions avoided. Among health outcomes, avoided inpatient hospitalizations contributed the most to avoided GHG emissions, followed by RSV-related primary care visits, A&E visits, primary care visits related to recurrent wheezing, ICU cases, and, lastly, follow-up consultant outpatient visits. Inpatient hospitalizations amounted to 9.0 kt CO_2_eq avoided in scenario 1 and 9.3 kt CO_2_eq avoided in scenario 2 (Table 2). For RSV-related primary care visits, an estimated 6.7 kt CO_2_eq (scenario 1) and 6.8 kt CO_2_eq (scenario 2) were avoided.

The sensitivity analysis provided a range of 16.3 to 23.8 kt CO_2_eq avoided in scenario 1 and 16.7 to 24.4 kt CO_2_eq avoided in scenario 2, with most variability in results attributable to the range in RSV-related hospitalizations (Table 2). Overall, each nirsevimab immunization avoided an equivalent of 31.0 [22.7; 34.2] kg CO_2_eq per infant in scenario 1 and 31.9 [23.3; 35.2] kg CO_2_eq per infant in scenario 2 at the use phase.

## Discussion

This study estimated that the emission avoidance benefit of a universal RSV immunization program with nirsevimab would be approximately 22 kt CO_2_eq each year in the UK and that approximately 30 kg CO_2_eq could be reduced along the patient care pathway per infant immunized. This is equivalent to approximately 35,000 return flights between London and New York for one person (39) or the CF of 14 million plastic bags (40). In the healthcare sector, this can be compared with five times the annual GHG footprint from operating theatres (41) or twice the annual fuel consumption from the London Ambulance Fleet Service (42).

The vast majority of avoided GHG emissions come from the reduction in RSV-related HCRU burden, particularly fewer hospitalizations. Since RSV is a leading cause of infant hospitalization, this intervention supports the mitigation of unnecessary carbon emissions in addition to freeing up capacity (16).

The one-dose schedule of nirsevimab versus the average four-dose schedule for palivizumab modeled in this study showed a significant reduction in GHG emissions; this demonstrates the ability of new immunization technologies to streamline the care pathway and, at the same time, reduce environmental impact, further demonstrating the importance of programmatic synergies in the assessment of new medicines.

The study reported in this manuscript focused its assessment on the “use” phase of the immunization by considering the treatment effect on the target population, to ultimately provide a complementary approach to the use phase analysis in “traditional” LCA. Comparing these data would allow for a firm indication of whether RSV prevention in infants with a long-acting mAb has a positive net impact on GHG emissions and could contribute to reducing the CF from the UK healthcare system, in addition to delivering tangible public health benefits. Furthermore, the integration of product-level LCA data for nirsevimab – once such data become available – could significantly enhance the environmental evaluation of its use. This would enable a more detailed understanding of its environmental impact across all stages of its life cycle. Incorporating this level of granularity would support more informed decision-making and contribute to a more comprehensive, system-wide perspective on the use of nirsevimab within healthcare settings.

The NHS has committed to deliver a “net zero” health service and the assessment of the environmental benefits of prevention programs with innovative therapeutics should be considered within this framework, along with patient and healthcare system benefits. Therefore, when mapping possible investments to reduce CF, ranging from better building insulation to electrifying the ambulance fleet, healthcare systems could also consider the GHG-intensity profile of new programs with innovative medicines. Optimizing care pathways while improving patient outcomes could be considered by healthcare systems to reduce GHG emissions and reach their environmental targets. This goes beyond traditional clinical and economic health technology assessment to encompass the measurement of the GHG intensity profile of new medicines from the patient care pathway perspective (43). Among other criteria, considering GHG emissions of technology interventions along the patient care pathway could be one of the key components of supporting sustainable quality improvement within HTAs (44;45). Currently, there is no standardized methodological guidance for incorporating environmental considerations of an intervention, such as nirsevimab, alongside clinical and economic outcomes. Environmental impact assessments should extend beyond the product’s life cycle emissions to also examine how the intervention affects the broader care pathway and contributes to the decarbonization of healthcare systems. Each health authority should consider integrating relevant environmental criteria in the context of their net-zero ambition and timeline.

The relationship between health and the environment is increasingly recognized and documented in the scientific literature, with climate change having multi-factorial public health consequences through air pollution, exposure to increasing temperatures, drought, food insecurity, and other mental and physical health risks (46). There has been a sense of urgency to tackle the environmental breakdown that contributes to poor human health and planetary health, reflected in current thinking on ways to include the environmental domain when conducting an HTA in Canada (47). Avoided GHG emissions from innovative medicines could be translated in terms of increased life years saved (LYS) or quality adjusted life years (QALYs), and ultimately be considered as part of cost-effectiveness evaluations (48). However, data on healthcare utilization emission factors remain scarce or not up-to-date, or are focused on a single healthcare outcome or limited to local case studies (27). Similarly, evidence on the link between greenhouse gases and public health impacts in terms of LYS or QALYs is limited, although increasingly documented (27). Further research in this field is needed.

This study has some limitations to be considered. First, the study omits other environmental impacts like water footprint or ecotoxicity and focuses solely on GHG emissions. The study does not include the GHG emissions associated with the development, manufacturing, distribution, and end of life of nirsevimab and palivizumab, or other activities that accompany drug interventions, like pharmacovigilance, activation of healthcare professionals, and promotional activities. No published LCA data were available at the time of the study for nirsevimab and palivizumab. To make the conclusion more robust, it would require direct comparison with LCA data for nirsevimab. However, to improve the environmental footprint of a clinical activity, the assessment should go beyond the product lifecycle measured by the manufacturer, requiring multi-stakeholder alignment between clinicians, hospital administrators, scientific societies, regulatory bodies, policy makers, and industry on what and how to measure care pathway emissions from healthcare delivery (44). The dual approaches of a product LCA and a patient care pathway assessment, from management of a disease and use of resources, could then be incorporated in the HTA evaluation.

The inputs used in the model are derived from a published cost-effectiveness model that relies on a set of assumptions on product efficacy, duration of protection, and uptake, among others.

## Conclusion

The novel approach to measuring the avoided GHG emissions of a drug intervention using a patient care pathway perspective, described here for RSV LRTI in infants, shows how immunization can provide environmental benefit to healthcare systems through improved patient outcomes. As part of the NHS effort to deliver net zero healthcare, environmental benefits from innovative preventative medicines should be considered from the patient care pathway perspective, in addition to evaluating the more traditional clinical and economic benefits; the GHG emissions cost of healthcare should be assessed with regard to health outcomes (9). To fully assess the impact of an intervention, further data describing the full product lifecycle is needed. Multi-stakeholder collaboration, exemplified by the SMI Health Systems Task Force initiative, will be instrumental in bridging data gaps, further exploring the potential environmental benefits of medicines, and aligning standards to report these at an industry level.

## Abbreviations

A&E: Accident and emergency
CF: Carbon footprint
CO_2_eq: Carbon dioxide equivalent
GHG: Greenhouse gas
GP: General practitioner
HCRU: Health care resource utilization
HTA: Health technological assessment
ICU: Intensive care unit
JCVI: Joint Committee on Vaccination and Immunisation
kg: kilogram
kt: Kiloton
LCA: Lifecycle assessment
LYS: Life years saved
LRTI: Lower respiratory tract infection
mAb: Monoclonal antibody
MHRA: Medicines and Healthcare products Regulatory Agency
NHS: National Health Service
NIP: National Immunisation Program
QALY: Quality-adjusted life year
RSV: Respiratory Syncytial Virus
S1: Scenario 1
S2: Scenario 2
SMI: Sustainable Markets Initiative
SoC: Standard of care
UK: United Kingdom
US: United States
WHO: World Health Organization
